# Projection surface detection and pose selection for autonomously displaying multimedia on walls using mobile robots

**DOI:** 10.3389/frobt.2026.1810071

**Published:** 2026-06-29

**Authors:** Kay Richter, Söhnke Benedikt Fischedick, Horst-Michael Gross

**Affiliations:** Neuroinformatics and Cognitive Robotics Lab, Ilmenau University of Technology, Ilmenau, Germany

**Keywords:** 3D NDTMap, human interface, mobile robot, projector, scene understanding, surface detection

## Abstract

Mobile robots equipped with projectors enable versatile applications such as multimedia display, interactive communication, and environmental augmentation. However, wall projection, which is required for displaying multimedia content on walls, remains challenging, because it is difficult to autonomously locate a projection space that is both flat and unobstructed. Some existing approaches address wall projection using 2D maps or by considering only large continuous surfaces, but these methods fail to capture the full potential of detailed 3D information of the operation area and often overlook wall segments that are partially occluded by objects, for example, areas blocked by pictures or whiteboards. To tackle these challenges, we propose a method based on 3D PanopticNDT maps, that uses semantic information to identify suitable wall segments. A tailored scoring function then evaluates potential robot poses to ensure optimal projection conditions, such as proper viewing angle, appropriate distance, and maximal surface area. Our experiments on both synthetic and real-world datasets demonstrate that our approach is well-suited for practical applications, effectively overcoming the limitations of earlier methods.

## 1 Introduction

**FIGURE 1:**
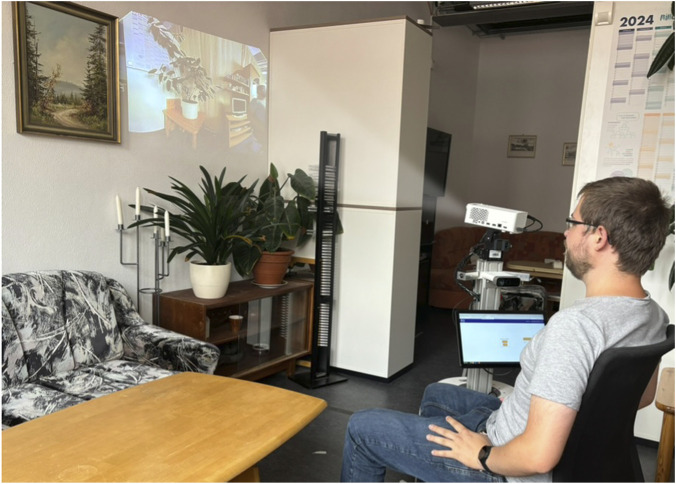
The robot autonomously identifies free wall space near the user and projects an image or a video stream. It avoids obstacles such as paintings to ensure that the projected content is fully visible. At the same time, the robot maintains visual contact with the user. This enables tasks such as video conferencing. The light beam connecting the projected image and the projector was added for improved visualization.

Mobile robots equipped with projectors provide a versatile platform for enhancing human-robot interaction and enabling novel applications. These include providing visual feedback on the robot intentions or status (Wengefeld et al., 2020), facilitating interactive games and entertainment (Park et al., 2018), and enabling a remote user to direct a robotic projector to light up specific objects in its environment (Machino et al., 2006), thereby facilitating communication and collaboration. For example, in our research project CO-HUMANICS we aim to leverage the potential of the projector attached to our robot, presented in Fischedick et al. (2023), for providing intention feedback, remotely highlighting objects of interest, and, as shown in Figure 1, autonomously projecting video calls or movies onto suitable surfaces within the environment, which is the focus of this paper.

However, autonomously identifying suitable projection surfaces with corresponding poses in a given environment is challenging. First, the projection surface must be flat to prevent distortion and maintain clarity of the projected content. Second, obstacles can obstruct the projection cone, possibly blocking the light from reaching the intended surface. Third, many projectors lack autofocus, relying on a fixed focal length–the robot must therefore maintain a specific distance to ensure sharp projections. Finally, the angle of the projection towards the wall should be considered, since a steep angle can reduce the quality of the projected images regarding focus, resolution and brightness. Addressing these factors is essential for effective robotic projection in real-world applications. While Stricker et al. (2015) and others already tackle these problems, i.e., by extracting wall segments from 2D Maps and show promising results, they do not consider 3D information, such as partial segments of the walls (e.g., wall above sofa) or partially occluded walls (e.g., by pictures or whiteboards) and thus can still be improved.

To tackle this challenge, in this paper, we propose an algorithm for autonomously identifying suitable robot poses, for projecting on visible surfaces. Our algorithm leverages PanopticNDT (Seichter et al., 2023) maps–a 3D room representation with semantic and instance labels–to find suitable walls as potential projection surfaces, by searching for flat straight wall segments. Together with the obstacles in the map, the identified surfaces are then used in a scoring function, to identify the optimal projection pose of the robot. The sampling of the scored poses can be combined with other criteria, such as staying in a specific room or proximity to a user, enabling applications like projecting a video call while maintaining an appropriate user distance. To evaluate the effectiveness of our proposed method, we conducted several experiments. As our approach heavily depends on the quality of the 3D maps, we leverage two distinct datasets to evaluate its performance under both ideal and non-ideal conditions. Given that our algorithm relies on high-quality 3D maps, we make use of the synthetic Hypersim (Roberts et al., 2021) dataset, which provides perfect depth measurements and ensures precise 3D PanopticNDT maps, thereby representing ideal mapping conditions. For real-world data, we used the ScanNet (Dai et al., 2017) dataset, offering realistic 3D maps of real environments, allowing us to evaluate our method’s performance under non-ideal conditions. Both datasets enable us to gather statistics, such as average projection surface size and distance. As both datasets are publically available, our proposed evaluation methodology can be used as a basis for other approaches to compare against our approach. Furthermore, we evaluated our proposed algorithm in seven real homes of elderly people, previously scanned as part of the MORPHIA project (Wengefeld et al., 2025), so that we could evaluate the accuracy in cluttered, realistic environments typical for our target application.

## 2 Related work

Projection systems have been widely explored in robotics for industrial (Andersen et al., 2016; Zaeh and Vogl, 2006; Xiang et al., 2021; Reinhart et al., 2007) and human-centered applications (Wengefeld et al., 2020; Stricker et al., 2015; Nakanishi, 2019; Watanabe et al., 2015; Takagi and Nakanishi, 2023; Izumi et al., 2011; Choi et al., 2013; Chadalavada et al., 2015; Fernández-Rodicio et al., 2017), with many systems using stationary projectors in controlled, static environments.

For example, Andersen et al. (2016) demonstrate stationary projection systems in industrial applications, where projectors remain fixed and serve tasks such as augmenting assembly processes or enhancing safety. While effective in their contexts, it lacks the flexibility and adaptability required for mobile robotic projection in dynamic or cluttered environments, a limitation our approach aims to address. Xiang et al. (2021) present a mobile robot in an industrial setting for collaborative tasks, where the robot should highlight pipes and infrastructure hidden in walls. This approach, while innovative, assumes AprilTag markers (Olson, 2011) and has no functionality for avoiding projection obstructions or dynamically tilting the projector to optimize for different surface angles.

In contrast to the industrial settings, many mobile projection systems focus on floor-based applications. Chadalavada et al. (2015), Nakanishi (2019), and Watanabe et al. (2015) propose approaches where mobile robots project paths onto the floor, serving as visual guides for humans interacting with the robots. Similar to that, Wengefeld et al. (2020) further explored floor projection for navigation and spatial augmented reality through a custom laser projection system. However, while achieving good results for providing intention feedback, those approaches do not target wall projections which are key components in our described interaction scenarios, particularly for media content, like video calls.

More directly related to our system are approaches using mobile projectors with some level of environmental interaction. Machino et al. (2006) proposed a remote-controlled robot equipped with a projector to highlight and annotate visible parts of the environment. However, this system lacks the ability for automatically identifying optimal projection surfaces, as they focus solely on remote controlling the robot. Stricker et al. (2015) developed a similar approach, utilizing 2D maps created by a 2D LIDAR Sensor, to automatically identify suitable projection surfaces. While this approach yields good results for environments with little furniture, it is not able to identify surfaces above furniture with a low height. For example, it can not identify the spaces above sofas or drawers as projection space. Additionally, similar to the other approaches, the projector is fixed at the robot, and can not be tilted independently. Fernández-Rodicio et al. (2017) presented an approach to identify projection surfaces by leveraging the information provided by a depth camera. This provides good results if there are good surfaces in view, but it is not able to find optimal projection poses within the environment, which ensure a high quality of the projection.

Another related research subject is projection mapping. It is goal is to augment reality by projecting computer generated graphics onto physical objects. Miyashita et al. (2018) for example, manages to project textures on dynamic surfaces to make them appear to be made out of different materials than they are. This work however, has no representation of its environment to identify suitable projections for flat imagery. Kasetani et al. (2015) utilize a mobile robot with a mounted projector to scan the surface of the object to map the projections on. While here the environment is mapped, it is not used to search for possible projection options, but to navigate the robot around the intended target object.

Our work addresses these limitations by introducing a fully autonomous approach for identifying suitable projection surfaces in 3D, while optimizing the robot’s projection pose through a novel scoring function. Unlike existing approaches we utilize 3D occupancy information as well as semantic labels, both provided by 3D PanopticNDT maps, which allow to solve fundamental problems found in previous work, such as finding flat surfaces in 3D, avoiding occlusion in the projection, and filtering non wall obstacles, including visual imagery like paintings or posters.

Additionally, none of the listed approaches considers applications in mobile, robot-assisted video conferencing, where both optimal projection surfaces and appropriate user-camera orientation are crucial. Our solution, therefore, presents advancements in enabling mobile robots to autonomously identify and project onto optimally suited wall areas in cluttered environments, while satisfying the requirements for video conferencing via the mobile robot.

## 3 Materials and methods

### 3.1 Proposed method

**FIGURE 2:**
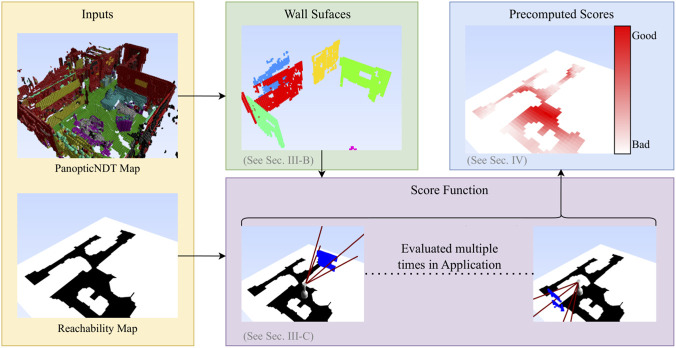
The presented approach takes a PanopticNDT map and a reachability map as inputs. The PanopticNDT map is preprocessed to identify the available projection surfaces. The score function uses these information to assign scores to poses in the environment, telling how good a pose is suited for projections. When applying the score function by brute force, scores for all poses can be precomputed.

Figure 2 shows our proposed method, which consists of multiple steps. In this section, we describe how we identify suitable projection surfaces and assign scores to 2D poses in the environment, while Section 3.2 demonstrates how these results are integrated into an application. Specifically, we begin with Section 3.1, where we briefly discuss the required input data for our approach. Building on that, Section 3.1 details how wall segments are extracted from the 3D PanopticNDT map (Seichter et al., 2023) to serve as candidate projection surfaces. Finally, Section 3.1 introduces our scoring function, which evaluates the suitability of a 2D pose within the environment for projection.

#### 3.1.1 Input data

Our approach relies on the PanopticNDT map, a comprehensive 3D representation that captures both the structural and semantic details of the environment. This rich model serves as the basis for extracting wall segments that are later evaluated for projection suitability.

In addition, our method requires a reachability map to ensure that only physically accessible projection poses are considered. For example, this prevents the selection of projection surfaces in isolated or unreachable rooms. To compute the reachability map, we preprocess a 2D occupancy map to identify positions that are accessible from the current robot pose. Dijkstra’s algorithm is employed from the robot’s location, assigning scores to reachable positions while marking inaccessible regions. This step is critical for efficiently filtering out invalid poses.

In summary, the precomputed PanopticNDT map, the computed reachability map, and the current robot pose together constitute the essential input data for our approach.

#### 3.1.2 Wall segment extraction

When trying to project images, it is important to know which flat surfaces of walls exist in the environment. Therefore, the first task of the preprocessing step is to identify a set of viable wall segments by leveraging the semantic information of PanopticNDT maps. An example for the input PanopticNDT map and the desired set of wall surfaces can be seen in Figure 2.

PanopticNDT maps provide, amongst other things, semantic labels for each cell of the 3D map, but do not explicitly capture complete wall surfaces. To extract continuous wall surfaces, we first collect cells recognized as the semantic class “wall” into a point cloud by only keeping the centers of the normal distributions and then iteratively identifying surfaces within. In each iteration, a flat wall model is fitted into the point cloud using RANSAC (Fischler and Bolles, 1981). Using this wall model, a set of points representing a piece of the wall is identified and all inliers of this model are removed from the point cloud, to prevent the same wall to be found again in following iterations. The peudocode for this process is shown in Algorithm 1 and more engineering details of our proposed wall extraction method are presented in Supplementary Appendix 1.1. As a result, we get a set of boxes $B_{\mathrm{other}}$ based on the voxels, which possibly obstruct the view to surfaces, and a list of identified walls segments $Walls=\left[\left(C_{\mathrm{thin,1}},B_{\mathrm{thick,1}}\right),\ldots ,\left(C_{\mathrm{thin,n}},B_{\mathrm{thick,n}}\right)\right]$ , where $C_{\mathrm{thin,i}}$ is a point cloud of the flat wall segment surface and $B_{\mathrm{thick,i}}$ is the corresponding list of obstacle boxes constructed of the same wall segment, possibly blocking the view onto other wall segments.

Algorithm 1 Preprocessing to identify walls surfaces.

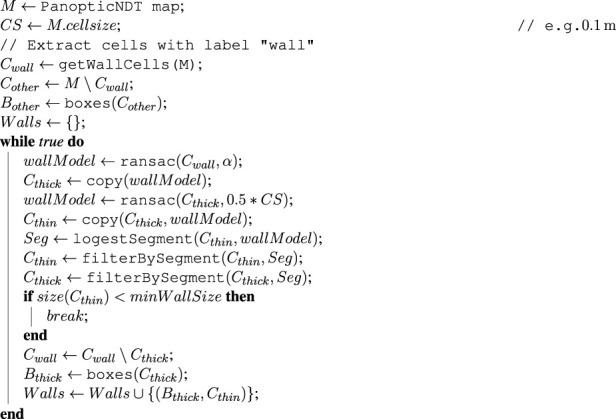

Note that in this step PanopticNDT maps or the line based RANSAC are exchangeable with other semantic 3D map representations and line identification algorithms. We decided to use PanopticNDT maps for their ability to be generated live and the efficient representation of the necessary data. However, any map that can be converted to semantically labeled points such as Sengupta and Sturgess (2015) and Alama et al. (2025) would suffice. We chose to apply RANSAC to identify walls withing the points, since it is a well understood and established algorithm.

#### 3.1.3 Score function for projection pose finding

To evaluate how well a given 2D pose $P_{\mathrm{base}}$ in the environment is suited for projection, we propose a score function, which computes a metric that quantifies the suitability of the pose for projection tasks. Note that this pose describes the orientation of the projector, which can be different from the orientation of the robot, since the projector can be panned using the pan-tilt unit, it is mounted on.

In application, the score function is invoked repeatedly during optimization (for example, in a brute-force search along the reachability map for the optimal projection pose). Given the high number of evaluations required, it is critical that the function operates efficiently. Therefore, the first step is to verify whether $P_{\mathrm{base}}$ is reachable by consulting the previously generated reachability map (see Section 3.1). If the pose is unreachable, the function skips the subsequent computations and assigns a score of 0.0. The next step is to identify the largest rectangle of the correct aspect ratio visible from $P_{\mathrm{base}}$ for each identified wall segment. This is done by first filtering out the points of $C_{\mathrm{thin,i}}$ , which are not visible to the projector using the obstructing boxes of $B_{\mathrm{other}}$ and $B_{\mathrm{thick,j}}\forall j\in \left[0,\ldots ,i-1,i+i,\ldots ,n\right]$ in combination with the projector intrinsics, and then searching for the largest fully visible rectangle on the remaining wall, resulting in four 3D coordinates, spanning the identified area. Supplementary Appendix 1.2 explains this step in detail. The area $a_{i}$ of this rectangle for each $C_{\mathrm{thin,i}}$ is then used as the basis for the score calculation.

The quality of a projection can be affected by both the angle and the distance between the projector and the target surface. To capture these effects, we adjust the base score $a_{i}$ accordingly.

First, because a steeper angle relative to the surface requires a stronger warp of the projected image to tackle distortion (as described in Section 3.2), and would therefore lower the quality, we modify $a_{i}$ using the following function:

$$scaleAngle\left(a_{i}\right)=a_{i}\cdot |sin\left(wall\text{\_}angle_{i}-pose\text{\_}angle\right)|$$

here, $wall\text{\_}angle_{i}$ represents the angle of the wall segment, while pose_angle is the angle of the line from $P_{\mathrm{base}}$ to the center of the projection surface within the map. This function scales $a_{i}$ to a factor of 1 at a right angle and to 0 at the steepest angle.

Furthermore, since our projector must be manually focused for a surface at a specific distance, deviations from this optimal distance $d_{\mathrm{optimum}}$ result in blurred projections. To account for this, we first calculate the distance $d_{\mathrm{rect,i}}$ from the center of the rectangle and then adjust the score using the following function:

$$scaleDist\left(a_{i}\right)=a_{i}\cdot \left(1.0-min\left(1.0,|\frac{\left(d_{\mathrm{rect,i}}-d_{\mathrm{optimum}}\right)}{d_{\mathrm{tolerance}}}|\right)\right)$$

here, $d_{\mathrm{tolerance}}$ describes the allowable deviation from the optimal distance. In case the projector has an auto-focus feature, the $scaleDist\left(a_{i}\right)$ can be simply set to 1, so it has no influence on the resulting score. These adjustments ensure that the final score reflects both the angular and distance-related factors affecting projection quality.

As mentioned before, each wall segment in Walls is scored individually, and only the highest score is yielded as the final score for the pose $P_{\mathrm{base}}$ . This can be expressed as:

$$\underset{i\in \left[0,\ldots ,n\right]}{\max}scaleDist\left(scaleAngle\left(a_{i}\right)\right)$$

This function rewards larger projection surfaces while penalizing deviations of the projection angles from the surface normal and distances that diverge from the preset focus.

### 3.2 Application

This section describes, how the previously presented algorithms can be integrated into a robot platform. Notice that the chosen mapping, localization, and navigation algorithms are independent of the proposed application. It covers all steps from an initial user interaction that triggers the process, to the moment the robot projects onto a selected surface.

The score function is highly versatile because it simply assigns a numerical value to each pose. For instance, it can be used to search for an optimal pose within a given radius or to ensure that the robot is oriented toward a specific surface, by evaluating only those poses that satisfy these predefined criteria. Once a user issues a command, the robot needs to find such candidate poses. Depending on the robot’s configuration, there are multiple options.

In the case where the PanopticNDT map and the occupancy map are generated once and remain static, a brute force approach can be applied. All scores for all possible poses within a grid are calculated in advance. When a user command is received, the system quickly evaluates only the poses that meet the specified criteria. The advantage of this precomputation is that no additional processing is required at runtime, resulting in a highly responsive user interaction. An example of these precomputed scores is shown in Figure 2, where the best score overall orientations is displayed for every evaluated position in the map.

However, if the mapping process is continuously running, the scores must be computed at runtime. In this dynamic scenario, heuristic approaches such as particle swarm optimization (PSO) can be employed. By using this method, poses are sampled based on the command issued by the user at the start of the process.

Both approaches yield a pose with the best score. Projecting an image also requires to determine the location of the surface in the room by, identifying the coordinates of its four corners. Therefore, we adapt the score function to return these corner points instead of a numerical score.

The robot then autonomously navigates to the selected pose using its navigation stack. Since the projector is mounted on a PTU, it can be directed toward the selected surface while still allowing the camera to be repositioned. This is especially useful if a local person should be kept in view. To avoid distortion in the projection, the projector’s camera parameters are used to back-project the corner points onto its two-dimensional image plane. These points are subsequently used to warp the media so that it fits exactly onto the rectangular projection surface, ensuring that the displayed image is properly aligned and free of distortion. This warping is performed using the OpenCV library (Bradski, 2000).

## 4 Results

Before presenting the experimental details, our experimental setup is described in Section 4.1. In the subsequent evaluation, we address three key aspects. First, in Section 4.2 we determine how often an external projection space at least two times larger than the built-in $15.1^{\prime \prime }$ display can be found. Next, in Section 4.3 we evaluate the average distance of the selected projection poses to establish an optimal search radius. This helps to determine a value, how far a robot needs to drive to project onto a suitable surface. Finally, in Section 4.4 we evaluate the quality of the surface detection in real-world maps.

### 4.1 Experiment setup

In our experiments, we use seven maps from the MORPHIA project (Wengefeld et al., 2025) along with data from the Hypersim (Roberts et al., 2021) and the ScanNet (Dai et al., 2017) datasets. The parameters used for the runs are listed in Table 1. Since the MORPHIA maps and the ScanNet contain considerable noise, the α parameter, discussed in Supplementary Appendix 1.1, is chosen to be accordingly higher than for the Hypersim dataset, which contains no noise. To apply our approach, these datasets are processed into the required input formats (see Figure 2). Note that the evaluation metrics presented later are independent of these preprocessing steps.

**TABLE 1:** Parameters used during evaluation.

Variable	Value
Noise handling parameter α	0.3 for MORPHIA
	0.3 for ScanNet
	0.1 for hypersim
Optimal distance $d_{\mathrm{optimal}}$ to projection surface	3.0 m
Allowed deviation $d_{\mathrm{tolerance}}$ from optimal distance	5.0 m
Minimum wall height for gap detection	0.5 m
Height of the projector	1.2 m

To achieve this, we first generate PanopticNDT maps for every scene in each dataset following the method described in Seichter et al. (2023). In addition to these 3D maps, we require corresponding occupancy maps, which we also retrieved from the PanopticNDT maps. Because the floor is not always positioned at the coordinate origin, we use the semantic annotation “floor” to determine the correct floor height. By combining the occupancy information from the PanopticNDT map with an offset of 0.1 m above the floor and a maximum height of 1.5 m similar to our mobile robot, we derive the required occupancy map.

In addition, a valid starting pose is needed for the robot to compute reachable areas. To this end, the robot is placed on a free cell within the occupancy map that is sufficiently distant from obstacles to avoid collisions. This starting pose is determined by finding the coordinate closest to the center of gravity of the semantic “floor” cells that does not intersect with any obstacles.

In all experiments, scores are computed for each map using a brute-force approach with a step size of 0.2 m and 36 uniformly spaced angles, corresponding to an angular step size of 0.1745rad. The measured average time over the MORPHIA dataset for a single pose is 0.015 s, proving applicability of the PSO approach in smaller indoor environments. However, in the current implementation, this time will grow exponentially with the size of the map, since every new obstacle cell is considered a potential obstruction for projecting onto any wall cell. The camera parameters of the projector are identical to those on our real robot, resulting in an aspect ratio of 16×9 and a projector height of 1.2 m. These parameters are used in the score function to derive the 3D projector pose $P_{\mathrm{projector}}$ from a sampled two-dimensional base pose $P_{\mathrm{base}}$ .

### 4.2 Minimum surface size

In our first series of experiments, we evaluate a scenario where a local user initiates wall projection. The user selects a search radius r around the robot within which the robot must operate to determine the optimal projection pose. A surface is considered valid if its diagonal is at least twice the size of the robot-mounted screen $\left(2\times 15.1^{\prime \prime }\right)$ . If this criterion is not met, the robot could simply move closer to the user, resulting in a better viewing experience.

**FIGURE 3:**
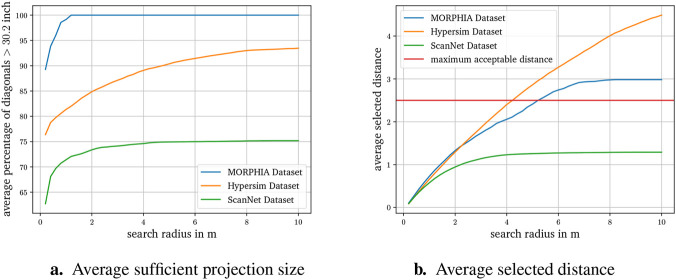
Results of experiments on the optimal search radius. **(a)** shows the percentage of poses where the detected wall area has a diagonal greater than $30.2^{\prime \prime }$ , while **(b)** shows the average distances of the selected projection poses within the search radius. For further details on these findings, we refer to Sections 4.2, 4.3.

To ensure representative data, we sampled 100 random reachable poses for each search radius, varying r from 0.2 m to 10.0 m in 0.2 m increments. Figure 3a shows the success percentage (i.e., large enough projection surface found) with respect to the search radius. The results show that the success percentage naturally increases with the search radius, as a larger area simply provides more opportunities to encounter a suitable projection surface. For example, the MORPHIA dataset achieves a success rate starting just below 90% and reaching 100% at a radius of 1.2 m. In contrast, the success rate for the ScanNet dataset approaches just above 75% as the radius increases. This difference may result from factors such as smaller available surfaces or navigable areas being located very close to walls, possibly due to significant noise in the depth measurements. The Hypersim dataset benefits the most from increasing radii, suggesting that these maps are larger and contain fewer walls within reach on average. While a search radius of 1.2 m yields satisfactory results on the MORPHIA dataset, the other datasets indicate that a slightly larger value may be preferable when using this function. Overall, the results indicate that, depending on the dataset, a sufficiently large projection surface can often be found within a reasonable distance.

### 4.3 Driven distance

In our scenario, it is important that the robot remains close enough to its user to capture the person with acceptable camera quality. For this reason, we aim for the robot to drive an average distance no greater than 2.5 m. Figure 3b shows the average selected distance as a function of the search radius. For the real-world maps from MORPHIA, the average distance approaches 3.0 m, while the chosen 2.5 m threshold is reached at a search radius of 5.0 m. This means that the robot can find a suitable projection space along the wall with a search radius of 5.0 m while still remaining within an acceptable distance to capture the local person. The ScanNet scenes appear to be smaller than those in the other two datasets, with the average distance converging to 1.3 m at a search radius of 10.0 m. In contrast, the Hypersim scenes, which are larger, benefit more from higher search radii, reaching the threshold at approximately 4.2 m. Overall, these results indicate that, in most cases, the presented approach provides satisfying results when a search radius between 2.0 m (for surface size) and 4.0 m (for camera visibility of the local person) is used.

### 4.4 Quality of found surfaces

**FIGURE 4:**
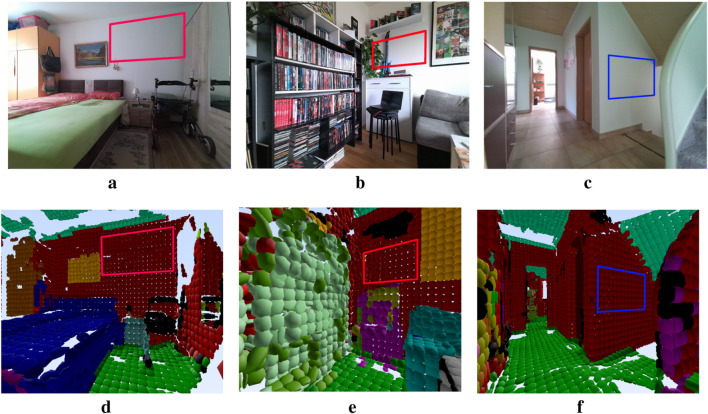
Examples of a true positive, false positive and false negative classifications in RGB images alongside the corresponding NDT-Map views. Red quadrangles indicate surfaces considered as suitable by the proposed approach. The quadrangle in the **(b)** is obstructed by an inflatable guitar that is not recognized as an obstacle in the NDT-Map (see **(e)**). The blue rectangle in image **(c)** highlights a white surface that would be suitable for projection but was not detected. **(a)** TP RGB. **(b)** FP RGB. **(c)** FN RGB. **(d)** TP NDT. **(e)** FP NDT. **(f)** FN NDT.

In a third experiment, we evaluate the surfaces detected by the proposed algorithm through human valuation of found projection surfaces on seven real-world maps. For this evaluation, surfaces from all poses in the brute force results with scores above the 0.5 median threshold were selected. Each of the three valuators reviewed the mapping process, overlaid with the detected projection surfaces onto the camera images, and classified the surfaces into true positives (TP), false positives (FP), and false negatives (FN) (see Figure 4). In particular, surfaces that overlap with obstacles by approximately 15% are counted as false positives. Since humans may have different views on the viability of a surface, the resulting count differs for each valuating person, resulting in an average of 28.5 viable projection surfaces over all seven maps. Based on these classifications, the approach achieves a recall of 73.6% , a precision of 79.2% , and an F1 score of 76.3% .

These results indicate that the algorithm is effective at identifying suitable projection surfaces. The recall suggests that a large majority of truly appropriate surfaces are correctly detected, while the precision implies that nearly four out of five identified surfaces are unobstructed and valid for projection. The balanced F1 score shows that the method maintains a good trade-off between detecting true surfaces and avoiding false detections. Overall, the performance metrics demonstrate that the proposed approach is reliable for finding projection surfaces in real-world environments.

## 5 Discussion

In this paper, we proposed an approach for automatically identifying suitable projection surfaces and determining corresponding poses for a mobile robot equipped with a mounted projector. Our method leverages PanopticNDT maps to detect flat wall surfaces and employs a scoring algorithm to evaluate candidate projection poses based on optimal angles, distances, and occlusion avoidance. The pose with the highest score not only serves as a reliable navigation goal but also provides the best available projection surface.

Experimental results show that our approach consistently finds adequate surfaces within the specified search radius, maintains proximity to the local user, and accurately identifies the majority of projection opportunities in real-world environments.

Even though the presented approach was developed for domestic indoor environments it is possible to apply the same principles in other environments such as industrial or outdoor settings, with the caveat that the PSO should not be used in larger environments, since the increasing number of obstacle cells in the map will lead to a significant slowdown. Besides the availability of suitable flat surfaces, one should also consider the longer distances necessary as discussed in Section 4.3. Finally, the semantic mapping algorithm should be adapted, to reliably identify walls in the targeted environment.

The current approach still has some weaknesses. When selecting the surface, the current lighting conditions are not considered, which can lead to a projection onto a surface already illuminated by a brighter source such as the sun. Also, the color if the selected wall is not integrated in the surface selection. In the worst case a black wall is selected, which would not reflect the projection as well as a white surface. Finally, only wall cells are considered as viable surfaces, even though it is possible for suitable surfaces to exist on larger furniture or doors.

In future work, we plan to extend this method to account for these mentioned shortcomings, and thereby further enhance the quality and applicability of the projected media. Also we plan to directly integrate the view of the human user watching the projection, by sampling only fitting poses or by adapting extending the score function to prefer surfaces in view.

## Data Availability

Publicly available datasets were analyzed in this study. This data can be found here: https://github.com/apple/ml-hypersim https://github.com/ScanNet/ScanNet.
