# Supplementary material for: Projection surface detection and pose selection for autonomously displaying multimedia on walls using mobile robots
Source: Front Robot AI. 2026 Jun 29;13:1810071. doi: 10.3389/frobt.2026.1810071 (PMC13358339; doi:10.3389/frobt.2026.1810071)
Supplement: Supplementary file 1 [file Supplementaryfile1.pdf]

# 1 APPENDIX

This section elaborates on the engineering details of the proposed approach. Sec. 1.1 explains the iterative process of identifying each wall segment in a PanopticNDT map. The process of identifying the largest visible area on a wall section for the projector and is explained in Sec. 1.2.

## 1.1 Wall Identification

This section explains the process of identifying walls segments in the PanopticNDT map in detail. First we collect the centers of the normal distributions of cells semantically labeled as *wall*, resulting in a sparse 3D point cloud, $C_{wall}$. The remaining occupied cells are converted into boxes of corresponding size and position and stored in $B_{other}$ for subsequent occlusion and visibility checks required for the scoring function. Although alternative representations – such as labeled point clouds or voxel maps with semantic annotations – can be used to generate $C_{wall}$ and $B_{other}$, these approaches are either memory and compute intensive or lack the required precision for accurately representing surface points.

As we are only interested in vertical walls, our goal of extracting wall segments is simplified. We begin by projecting the point cloud $C_{wall}$ along the vertical axis to obtain a top-down 2D representation. In this projection, vertical walls appear as dense, linear clusters of points, which can be effectively detected using RANSAC Fischler and Bolles (1981) to fit a line model. Tilted walls, being less densely represented in the projection, are less likely to be detected. As a byproduct of fitting a line model, there is no need to explicitly handle wall corners since these regions are typically undesirable as projection surfaces due to distortion, as the process naturally excludes them. Choosing an appropriate inlier distance $\alpha$ is critical, as localization errors and sensor noise may have caused walls in the constructed map to be thicker than a single cell, requiring a corresponding adjustment of $\alpha$. The line model obtained from RANSAC is then used to construct a point cloud

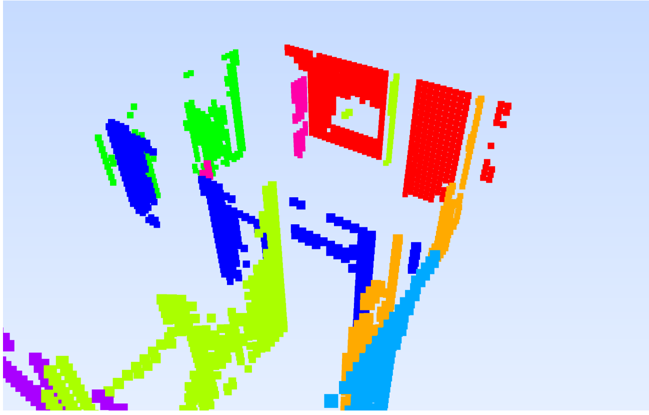

**Figure S1.** Resulting walls detected, if $C_{thin}$ is not reduced to the longest connected segment. The red wall is split into two parts by the lime wall, since the lime wall was identified in an earlier iteration. This can be avoided by only keeping the longest connected segment of a wall during each iteration.

$C_{thick}$, comprising all inlier cells corresponding to the identified wall. If $\alpha$ is too low, $C_{thick}$ may not contain all cells belonging the same wall, which will allow the remaining cells to be selected as a different wall during the remaining iterations, and therefore obstructing each other. On the other hand, if $\alpha$ is too high, the identified lines may not follow the real walls, resulting $C_{thick}$ containing parts of multiple walls.

To reduce the processing load for the subsequent scoring function and to obtain a more accurate representation of the wall by mitigating mapping errors, we further refine $C_{thick}$ into a point cloud that represents the wall with a single cell thickness. This refinement is achieved by applying RANSAC once again to $C_{thick}$ using an inlier distance set to half the cell size (i.e. $0.5 \cdot CS$) of the input PanopticNDT map. The resulting line model is then used, as in the previous step, to generate a new point cloud $C_{thin}$.

At this stage, the wall representation is sufficient in terms of tilt and thickness. However, since the line models found by RANSAC are infinitely long, the derived point clouds also contain all cells crossing these models. This can result in large gaps at doors or rooms and may include parts of walls perpendicular to the found line

as shown in Fig. S1. To address this issue, we retain only the longest connected segment of the wall for the current iteration. To identify this segment, the point cloud $C_{thin}$ is projected onto the primary direction of its 2D line model, and the projected points are binned according to the cells of the source PanopticNDT map. This process produces an array that represents the number of wall cells encountered along the wall's length. A threshold – derived by dividing the desired minimum height of a wall surface by the cell size of the PanopticNDT map – is applied to distinguish gaps from continuous wall segments. Using this criterion, the longest valid section of the wall is identified. The resulting segment, denoted as $Seg$, is then used to trim both $C_{thick}$ and $C_{thin}$, ensuring that no points outside the detected borders along the model line are retained.

The final step in each iteration is to verify whether the thin wall $C_{thin}$ is sufficiently large to be considered a valid projection surface by counting the number of its contained points. If the segment meets the required size, all points in $C_{thick}$ that belong to the detected wall segment are removed from $C_{wall}$ to prevent their re-detection in subsequent iterations. The wall point clouds are then restored to three dimensions before $C_{thick}$ is converted into a list of boxes $B_{thick}$ (analogous to $B_{other}$) and combined with $C_{thin}$ into the overall set of detected walls, denoted as $Walls$. If, however, the wall segment is too small, the algorithm terminates. As a result, our algorithm yields a list of obstacles $B_{other}$ and a set of $n$ wall surfaces $Walls$, where each wall surface is represented as a pair consisting of the obstacle representation $B_{thick,i}$ and the corresponding refined flat wall surface $C_{thin,i}$.

### 1.2 Pose Scoring

As a basis for the score calculation, we use the largest fully visible rectangles from the previously identified wall segments. To obtain these, we filter each point cloud $C_{thin,i}$ by evaluating their visibility. This process requires the 3D pose of the projector $P_{projector}$, which we compute from the two-dimensional input pose $P_{base}$ and the known translation of the projector to the base. With the projector intrinsics available, visibility checks are skipped for points that fall outside the projector's view cone. Since $P_{base}$ is two-dimensional, the derived $P_{projector}$ only determines a pan direction for the projector's pan-tilt unit and does not specify a tilt angle. Therefore, we only bypass visibility checks for points to the left and right of the view cone, while points above and below are still evaluated. The visibility of each point is determined by testing whether a ray from $P_{projector}$ to the point intersects any obstacle boxes. All points in a wall segment $C_{thin,i}$ share the common obstacles $B_{other}$ as well as the obstacles from all other wall segments $B_{thick,j} \forall j \in [0, \cdots, i-1, i+1, \cdots, n]$.

At this stage, we compute a score for each wall segment and return the highest score as the score for the input pose $P_{base}$. The process begins by identifying the largest rectangle with the desired aspect ratio contained within the visible surface of each wall segment. Since verifying the visibility of neighboring cells in a 3D point cloud is complex, the point cloud is first transformed into a two-dimensional grid by projecting each point along the x-axis or y-axis, depending on the wall's primary direction of its 2D line model (see Fig. S2). Since each point originally stems from a voxel, we can easily construct a grid from this projection. Each cell in this grid records its corresponding 3D coordinates and whether a wall point is present. This data structure simplifies the task of finding the largest visible rectangle with the specified aspect ratio. The area $a_i$ of this rectangle for each $C_{thin,i}$ is then used as the basis for the score calculation.

## REFERENCES

Fischler, M. A. and Bolles, R. C. (1981). Random sample consensus: a paradigm for model fitting with applications to image analysis and automated cartography. *Commun. ACM* 24, 381–395. doi:10.1145/358669.358692

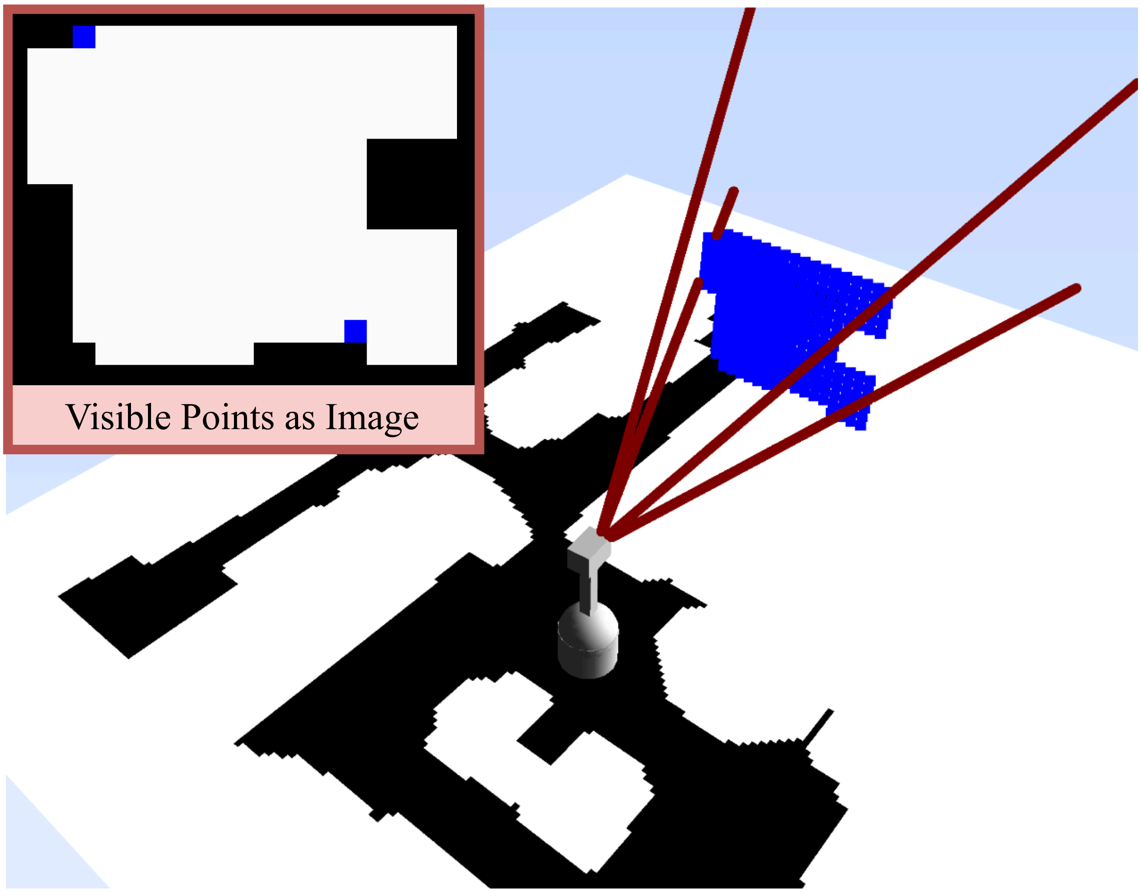


**Figure S2.** Result of filtering all non-visible cells for pose. Top left shows how these cells are projected along the y-axis into a 2D image. White = cell in wall exists; Black = cell in wall is missing; Blue = corners of the rectangle containing the largest rectangle of given aspect ratio
